# A Review on Central Nervous System Effects of Gastrodin

**DOI:** 10.3389/fphar.2018.00024

**Published:** 2018-02-02

**Authors:** Yuan Liu, Jialiang Gao, Min Peng, Hongyan Meng, Hongbo Ma, Pingping Cai, Yuan Xu, Qiong Zhao, Guomin Si

**Affiliations:** ^1^Department of Traditional Chinese Medicine, Shandong Provincial Hospital Affiliated to Shandong University, Jinan, China; ^2^School of Traditional Chinese Medicine, Shandong University of Traditional Chinese Medicine, Jinan, China; ^3^Department of Cardiology, Guang'anmen Hospital, China Academy of Chinese Medical Sciences, Beijing, China

**Keywords:** *Gastrodia*, *Gastrodia elata* Blume, Chinese medicine, pharmacology, pharmacokinetics, central nervous system disorders

## Abstract

*Rhizoma Gastrodiae* (also known as *Tian ma*), the dried rhizome of *Gastrodia elata* Blume, is a famous Chinese herb that has been traditionally used for the treatment of headache, dizziness, spasm, epilepsy, stoke, amnesia and other disorders for centuries. Gastrodin, a phenolic glycoside, is the main bioactive constituent of *Rhizoma Gastrodiae*. Since identified in 1978, gastrodin has been extensively investigated on its pharmacological properties. In this article, we reviewed the central nervous system (CNS) effects of gastrodin in preclinical models of CNS disorders including epilepsy, Alzheimer's disease, Parkinson's disease, affective disorders, cerebral ischemia/reperfusion, cognitive impairment as well as the underlying mechanisms involved and, where possible, clinical data that support the pharmacological activities. The sources and pharmacokinetics of gastrodin were also reviewed here. As a result, gastrodin possesses a broad range of beneficial effects on the above-mentioned CNS diseases, and the mechanisms of actions include modulating neurotransmitters, antioxidative, anti-inflammatory, suppressing microglial activation, regulating mitochondrial cascades, up-regulating neurotrophins, etc. However, more detailed clinical trials are still in need for positioning it in the treatment of neurological disorders.

## Introduction

A central nervous system (CNS) disorder refers to a disease that affects the structure or function of brain or spinal cord, thus causing neurological or psychiatric complications (Kundap et al., 2017). In recent decades, the number of people suffering from CNS disorders has grown dramatically due to the increase of life expectancy, placing a tremendous burden on families and social economies (Alavijeh et al., 2005). However, although much progress have been made in understanding the underlying mechanisms of these disorders, there are no effective therapy to prevent or stop the progression of these conditions (Upadhyay, 2014), and the existing drugs have only symptomatic effects as well as many adverse reactions, calling for a strong need for identifying novel therapeutic opportunities (Kazantsev and Outeiro, 2010). In this context, herbal plants and their bioactive compounds have drawn much attention in their potential as new treatment options for CNS disorders (World Health Organization, 1999). Actually, several compounds with strong actions derived from herbs, such as cannabidivarin, cannabidiol, and huperzine A, have recently been developed as drug candidates in western countries (Bialer et al., 2015).

**Figure 1:**
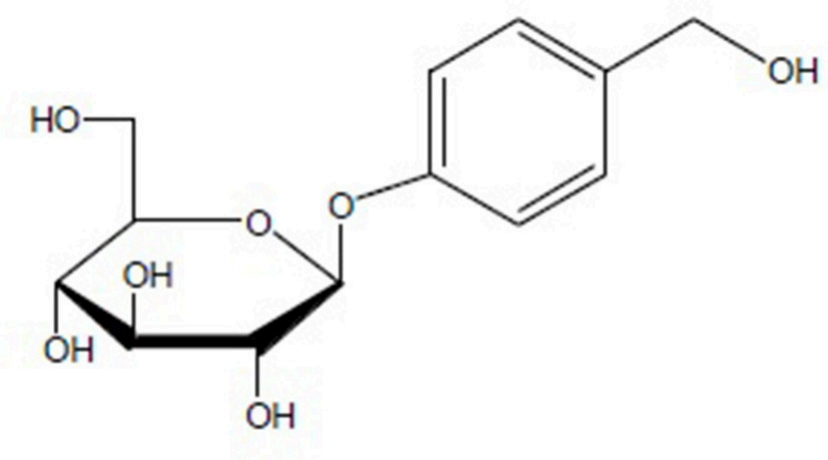
The chemical structural formula of Gastrodin.

*Gastrodia elata* Blume (*G. elata*) is a notable herbal plant that has been traditionally used to treat various conditions including headache, dizziness, spasm, epilepsy, stoke, amnesia, and other disorders in oriental countries for centuries (Chinese Pharmacopoeia Commission, 2015). The main medicinal part is its rhizome, called *Rhizoma Gastrodiae*. Since 1950s, over 81 compounds have been isolated from *G. elata* including phenolics, polysaccharides, organic acids, sterols, etc. (Duan et al., 2013). Gastrodin (PubChem CID: 115027), a phenolic glycoside that chemically known as 4-hydroxybenzyl alcohol-4-*O-*β*-D*-glucopyranoside, is the second compound identified from the plant after vanilyalcohol, which was isolated in 1958 (Liu and Yang, 1958), and is considered to be the main bioactive constituent of *Rhizoma Gastrodiae*. Furthermore, the content of gastrodin is assayed as the most important phytochemical marker in the quality standardization of *Rhizoma Gastrodiae* (Tao et al., 2009). With a molecular formula of C_13_H_18_O_7_ and a molecular weight of 286 Da, gastrodin is easy to dissolve in methanol, ethanol and water, while it is difficult to dissolve in chloroform and ether. The chemical structural formula of gastrodin is shown as Figure 1 (Zhou et al., 1979).

In 1978, two laboratories simultaneously reported the isolation of gastrodin along with other six compounds from the ethanolic extract of *G. elata* (Feng et al., 1979; Zhou et al., 1979). Since then, gastrodin have been extensively investigated on its biological actions. As a result, numerous pharmacological activities have been attributed to gastrodin, including sedative, hypnotic (Deng and Mo, 1979), anti-vertigo (Chen et al., 2004), analgesic (Zhang et al., 2006), anti-epileptic (Ojemann et al., 2006), antidepressant (Chen and Sheen, 2011), anxiolytic (Peng et al., 2013), memory-improving (Hsieh et al., 1997), anti-aging (Wang Z. J. et al., 2007), lowering blood pressure (Zhang Q. et al., 2008), preventing osteonecrosis (Zheng et al., 2014) effects, etc. Among its various pharmacological properties, its strong actions in CNS diseases appear to be particularly prominent. Considering its low toxicity and remarkable pharmacological performance, gastrodin might be a potential valuable therapeutic for the prevention and treatment of some CNS disorders (Zhan et al., 2016).

**Figure 2:**
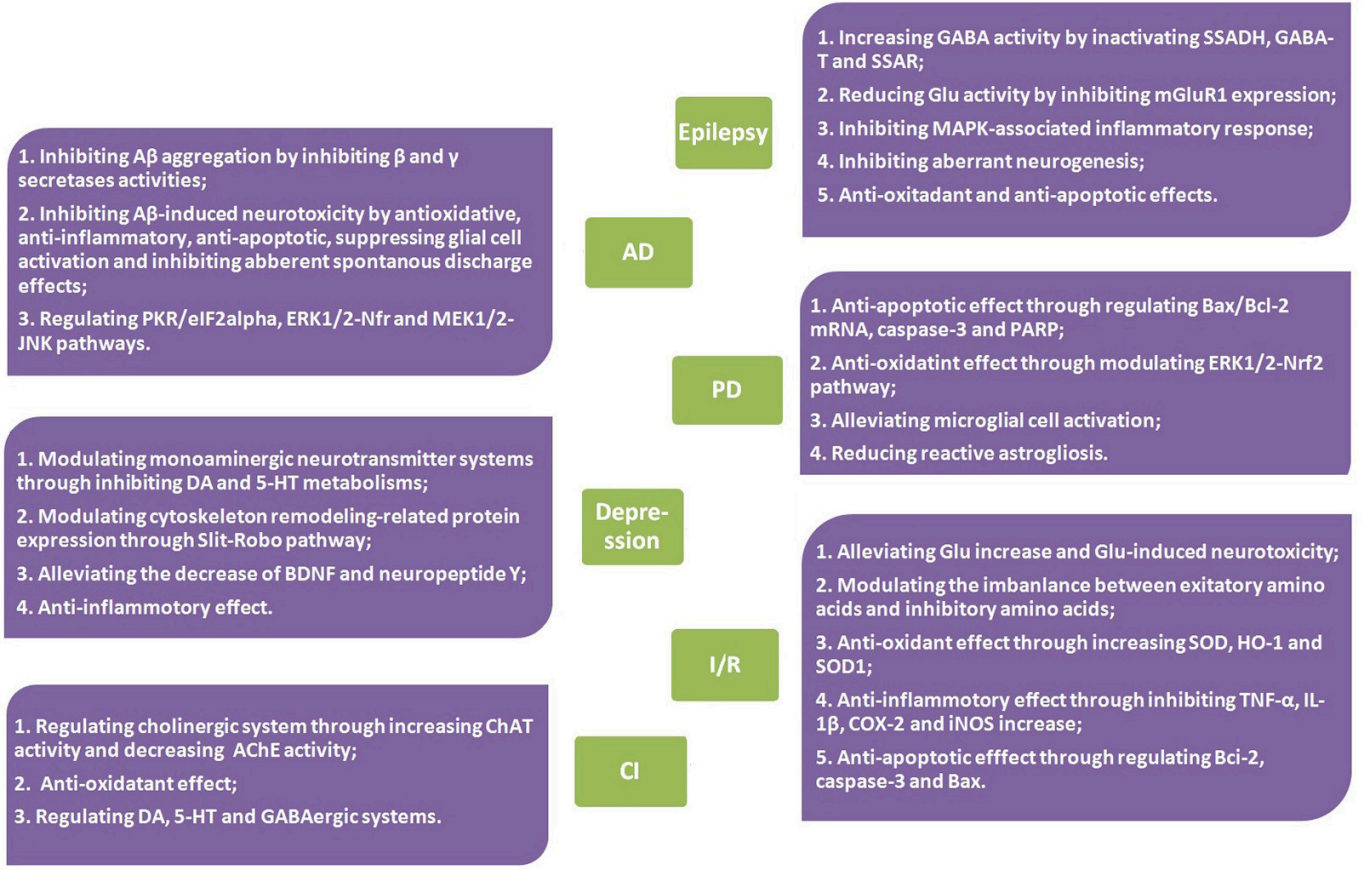
Mechanisms of action of gastrodin in CNS disorders.

However, up to now, although several reviews on *G. elata* have been conducted (Chen and Sheen, 2011; Jang et al., 2015; Matias et al., 2016; Zhan et al., 2016), in which gastrodin and its therapeutic effects were sporadically mentioned, the pharmacological properties of gastrodin has not been systematically reviewed. In this article, focusing on the neuropharmacological properties of gastrodin, the therapeutic effects of gastrodin in preclinical models of CNS disorders including epilepsy, Alzheimer's disease, Parkinson's disease, affective disorders, cerebral ischemia/reperfusion, and cognitive impairment were reviewed (Tables 1, 2) as well as the underlying mechanisms involved (Figure 2) and, where possible, clinical data that support therapeutic effects (Table 3). In addition, the sources, pharmacokinetics, and toxicity of gastrodin were also reviewed here.

**Table 1:** Effects of Gastrodin in *in vivo* models of epilepsy.

**Model^a^**	**Animal**	**Dosage of gastrodin^b^**	**Major findings^c^**	**References**
PTZ-induced acute seizures	Mice	125 mg/kg, *i.p*.	↑Seizure latency	Deng and Mo, 1979
Coriaria Laetone-induced acute seizures	Rabbits	1,000, 2,000, 3,000 mg/kg, *i.v*.	↑EEG performance, ↑seizure latency, ↓seizure incidence rate,↓seizure duration, ↓seizure severity, ↓mortality rate, ↓recovery time	Chai et al., 1983
Genetic seizure-sensitive gerbils induced by rigorous stoking of the back	Gerbils	60 mg/kg/d for 7 d, *p.o*.	↓Seizure score; ↓SSADH, ↓GABA-T, ↓SSAR, ±GAD65, ±GAD67	An et al., 2003
PTZ-kindling chronic epileptic seizures	Male SD rats	100 mg/kg/d for 28 d, *i.p*.	↓Seizure score, ↑EEG performance; ↓GAP-43 expression	Lian et al., 2005
PTZ-kindling chronic epileptic seizures	Wistar rats	50, 100 mg/kg/d for 28 d, *i.g*.	↓Seizure incidence rate, ↓seizure score; ↓Cx43, ↓synaptophysin	Cao et al., 2008a,b
PTZ-induced acute seizures	Male Wistar rats	30 mg/kg, *i.p*.	↓Seizure grade; ↓Glu, ↑GABA	Chen and Tian, 2009
PTZ-kindling chronic epileptic seizures	Male Wistar rats	100, 200 mg/kg for 7 d, *i.p*.	↑Seizure latency, ↓seizure duration, ↓seizure intensity; ↓mGluR1, ↓PKCα	Mu et al., 2009
PTZ-kindling chronic epileptic seizures	Wistar rats	200 mg/kg for 10 d, *i.p*.	↓Seizure score, ↑EEG performance; ↑SOD, ↑GSH-Px, ↓MDA, ↓p38 MAPK, ↓IL-1β, ↓IL-2, ↓IL-6, ↓TNF-α	Li and Cheng, 2015; Li et al., 2015
Li-pilo-induced acute seizures	Male SD rats	60, 90, 120 mg/kg, *i.p*	↓Seizure score; ↑CAT, ↑GSH, ↑SOD, ↑GR, ↑T-AOC, ↓MDA, ↓p38 MAPK	Zhong et al., 2016
Li-pilo-induced acute seizures	Male SD rats	60, 120, 180 mg/kg, *i.p*.	↓Seizure score, ↑seizure latency, ↓mortality rate; ↑Bcl-2, ↓caspase-3	Bian et al., 2016
Li-pilo-induced status epilepticus	SD rats	100 mM (5 μl), *i.c.v*.	±Seizure severity, ±seizure duration, ±mortality rate	Wong et al., 2016
Li-pilo-induced status epilepticus	Male SD rats	10 mM/d for 9 d, *i.c.v*.	↓Seizure incidence rate, ↑seizure latency, seizure score, ↑EEG performance; ↓EC neuronal death, ↓EC neuronal discharge, ↓Nav 1.6 currents, ↓Nav 1.6 expression	Shao et al., 2017
PTZ-induced acute seizures	Male C57BL/6 mice	50, 100, 200 mg/kg, *i.p*.	↑Seizure latency, ↓seizure intensity, ↑EEG performance; ↓IL-1β, ↓TNF-α, ↓NF-κB, ↓MAPK, ↓CREB	Chen et al., 2017

a PTZ, pentetrazol; Li-pilo, lithium-pilocarpine;

b i.p., intraperitoneal administration; i.v., intravenous administration; p.o., oral administration; i.g., intragastric administration; i.c.v., intracerebroventricular injection.

c *↑, increase significantly; ↓, decrease significantly; ±, no significant effect*.

**Table 2:** Effects of Gastrodin in *in vivo* and *in vitro* models of AD, PD, anxiety, depression, I/R, VD and cognitive impairment.

**Model^a^**	**Type**	**Inducer^b^**	**Animal/cell^c^**	**Major findings^d^**	**References**
AD	*In vitro*	Aβ_25*-*35_	Rat cortical and hippocampus cells	↑Cell survival, ↓LDH release	Liu et al., 2005
AD	*In vitro*	–	293sw and APP695 transgenic cells	↑Cell viability, ↑stability of membranes; ↓APP, ↓Aβ; ±IDE levels	Liu et al., 2011
AD	*In vitro*	Aβ_1*-*42_	BV2 mouse microglial cells	↑Cell viability; ↑GRP78, ±CHOP	Lee et al., 2012
AD	*In vitro*	Aβ_1*-*42_	Rat hippocampal neurons	↑Cell viability; ↑CAT, ↑SOD, ↑Nrf2, ↑ERK1/2	Zhao et al., 2012
AD	*In vivo*	Aβ_1*-*40_	Male SD rats	↑Learning/memory ability	Liu and Wang, 2012
AD	*In vivo*	Aβ_1*-*40_	Tree shrews	↑BDNF	He et al., 2013
AD	*In vivo*	Aβ_1*-*42_	Male SD rats	↓EC neuronal activities	Chen Y. Y. et al., 2014
AD	*In vivo*	–	Tg2576 transgenic mice	↑Memory ability; ↓Aβ deposition; ↓Iba-1, ↓GFAP	Hu et al., 2014
AD	*In vitro*	–	117 and 146 transgenic cells	↑Cell viability; ↓extracellular and intracellular Aβ, ↓β-secretases, ↓γ-secretases	Zhu et al., 2014
AD	*In vivo*	–	J20 and 5XFAD transgenic mice	↑Learning/memory ability; ↓Aβ level, ↓Aβ deposition	Zhu et al., 2014
AD	*In vitro*	Aβ_1*-*42_	Rat progenitor cells	↑Cell viability; ↓TNF-α, ↓IL-1β, ↓IL-6, ↓NO, ↓iNOS, ↑COX-2, ↑Bcl-2, ↓Bax, ↓MEK1/2, ↓ERK, ↓JNK, ±p38 MAPK	Li and Qian, 2016
AD	*In vivo*	Aβ_1*-*42_	C57BL/6 mice	↑SOX-2, ↑DCX	Li and Qian, 2016
AD	*In vivo*	–	Tg2576 transgenic mice	↑Learning/memory ability; ↓β-secretases, ↑SOD, ↑CAT, ↓MDA, ↓ROS, ↓PKR, ↓elF2alpha	Zhang et al., 2016
AD	*In vivo*	–	5XFAD transgenic mice	↑Learning/memory ability; ↓Aβ level, ↓Aβ deposition	Zhou et al., 2016
AD	*In vitro*	–	117 transgenic cells	↑Cell viability; ↓extracellular and intracellular Aβ levels, ↓β-secretase	Zhou et al., 2016
PD	*In vivo*	Rotenone	Male Wistar rats	↓Motor deficits; ↓ DA neuron loss, ↑TH-positive cells, ↓IL-1β, ↓TNF-α	Li et al., 2012
PD	*In vitro*	MPTP	Human DA SH-SY5Y cells	↑Cell viability; ↓free radicals, ↓ROS, ↑SOD, ↑Bcl-2, ↓Bax, ↓Caspase 3, ↓PARP cleavage	Kumar et al., 2013
PD	*In vivo*	MPTP	Male C57BL/6 mice	↓Motor deficits, ↓bradykinesia; ↑TH, ↑GFAP	Kumar et al., 2013
PD	*In vivo*	6-OHDA	Male SD rats	↓Motor deficits; ↑TH-positive neuron	Wang et al., 2013
PD	*In vivo*	Rotenone	Lewis rats	↓Cx43	Wang et al., 2013
PD	*In vitro*	Rotenone	Rat astrocytes	↓Cx43, ↓GJIC	Wang et al., 2013
PD	*In vivo*	MPTP	Male C57BL/6 mice	↓Motor deficits; ↓MDA, ↑HO-1, ↑SOD, ↑GSH, ↑ERK1/2, ↑Nrf-2	Wang H. et al., 2014
PD	*In vivo*	6-OHDA	Male SD rats	↓Motor deficits; ↑Bcl-2, ↓Bax	Xi and Ren, 2016
Anxiety	*In vivo*	ESPS	Male SD rats	↓Anxiety-like behaviors (in OFT, SPT); ↓IL-1β, ↓IL-6, ↓iNOS, ↓p38 MAPK	Peng et al., 2013
Depression	*In vivo*	CUS	Male SD rats	↓Depression-like behavior (in SPT, FST and MWM); ↑NSCs proliferation, ↓p-iκB, ↓NF-κB, ↓IL-1β	Wang H. et al., 2014
Depression	*In vivo*	CUS	Male SD rats	↓Depression-like behavior (in SPT and FST); ↑GFAP, ↑BDNP	Zhang et al., 2014
Depression	*In vivo*	FST	Male SD rats	↓Depression-like behavior (in FST); ↑5-HT1A receptor expression, ↓5-HIAA/5-HT, ↓(DOPAC+HVA)/DA, ↓Slit1, ↓RhoA, ↑CRMP2, ↑PFN1	Chen et al., 2016
Depression	*In vivo*	SPS	Male SD rats	↓Depression-like behavior (in FST); ↓NE, ↓TH, ↑neuropeptide Y, ↑BNDF	Lee et al., 2016
Depression	*In vivo*	UCMS	Male SD rats	↓Depression-like behaviors (in OFT and SPT); ↓5-HIAA/5-HT, ↓(DOPAC+HVA)/DA, ↓corticosterone	Lin et al., 2016
Depression	*In vivo*	CUS	Male SD rats	↓Depression-like behaviors (in FST and SPT); ↓IL-1β, ↓IL-6	Sun et al., 2017
I/R	*In vitro*	Simulated I/R environment	Rat cortical neurons	↓LDH release, ↑membrane fluidity; ↓LPO	Xue et al., 1998
I/R	*In vitro*	Simulated I/R environment	Rat astrocytes	↓LDH release; ↓GFAP, ↓NOS	Hu et al., 2001
I/R	*In vivo*	MCAO	Male SD rats	↑Neurological function, ↓infarct volume, ↓edema volume; ↓Glu, ↓Glu/GABA	Zeng et al., 2006, 2007
I/R	*In vitro*	OGD	Rat hippocampal cells	↑Cell viability; ↓extracellular Glu level, ↓Ca^2+^ overload, ↓NO	Zeng et al., 2006, 2007
I/R	*In vivo*	MCAO	Male SD rats	↓Infarct volume; ↓Glu, ↓Asp, ↓GABA, ↓Tau, ↓Glu/GABA	Bie et al., 2007
I/R	*In vivo*	MCAO	Male SD rats	↓Infarct volume; ↓TUNNEL-positive neurons, ↓Caspase-3	Nie et al., 2012
I/R	*In vivo*	MCAO	Male SD rats	↑Neurological function, ↓infarct volume, ↓edema volume; ↓IL-1β, ↓IL-6, ↓TNF-α	Li et al., 2014
I/R	*In vivo*	MCAO	C57BL/6 mice	↓Infarct volume, ↑neurological function; ↓casapase-3, ↓Bax, ↑Bcl-2, ↑SOD, ↓MDA, ↑HO-1, ↑Akt, ↓TNF-α, ↑Nrf-2,↓IL-1β	Peng et al., 2015
I/R	*In vivo*	MCAO	Male SD rats	↑Neurological function, ↓infarct volume; ↓TUNNEL-positive neurons, ↓IL-1β, ↓COX-2, ↓iNOS, ↓Caspase-3	Liu et al., 2016
VD	*In vivo*	MCAO	Male SD rats	↑Learning/memory ability; ↓AChE, ↑ ChAT, ↓Glu	Zhang L. D. et al., 2008
VD	*In vivo*	2-VO	Male Wistar rats	↑Learning/memory ability; ↓MDA, ↑GSH-Px, ↑total thiol	Li and Zhang, 2015
CI	*In vivo*	SCOP	Male SD rats	±Memory impairment	
CI	*In vivo*	CXM	Male SD rats	↓Memory impairment	
CI	*In vivo*	APO	Male SD rats	↓Memory impairment	Hsieh et al., 1997
CI	*In vivo*	IDPN	Male Wistar rats	↑Memory function; ↑DA, ↑HVA, ↑DOPAC, ↓ (DOPAC+HVA)/DA, ↓DA D_2_ receptor, ↑DAT	Wang X. et al., 2014
CI	*In vivo*	IDPN	Male Wistar rats	↑Memory function; ↑5-HT, ↓5-HIAA/5-HT, ↓SERT, ↑5-HT1A receptor	Wang et al., 2015
CI	*In vivo*	IDPN	Male Wistar rats	↑Learning/memory ability; ↑GABA, ↓a2 GABA_A_ receptor	Wang et al., 2016

a AD, Alzheimer's disease; PD, Parkinson's disease; I/R, ischemia/reperfusion; VD, vascular dementia; CI, cognitive impairment.

b Aβ, amyloid beta; 6-OHDA, 6-hydroxydopamine; MPTP, 1-Methyl-4-phenyl-1,2,3,6-tetrahydropyridine; ESPS, enhanced single prolonged stress; CUS, chronic unpredictable stress; FST, forced swimming test; SPS, single prolonged stress; UCMS, unpredictable chronic mild stress; MCAO, middle cerebral artery occlusion, OGD, oxygen/glucose deprivation; 4-VO, 4-vessel occlusion; 2-VO, 2-vessel occlusion; SCOP, scopolamine; CXM, cycloheximide; APO, apomorphine; IDPN, 3,3′-iminodipropionitrile.

c 293sw and APP695 transgenic cells, transgenic cells that over-express APP; Tg2576 transgenic mice, transgenic mice that over-express APP; 117 transgenic cells, transgenic cells that over-express Aβ and β secretase; 146 transgenic cells, transgenic cells that over-express Aβ and γ secretase; J20 transgenic mice, transgenic mice that overexpress APP; 5XFAD transgenic mice, transgenic mice that overexpress APP/PS1.

d *↑, increase significantly; ↓, decrease significantly; ±, no significant effect*.

**Table 3:** Clinical trials performed of gastrodin for CNS disorders.

**Target^a^**	**Design^b^**	**Dose regimen^c^ (Case/control)**	**Duration**	**Case/control**	**Main outcomes^d^**	**Side effects**	**References**
Chronic refractory epilepsy	Case series	Gastrodin (100 mg, *p.o*., tid) plus original AEDs/-	12 weeks	15/-	Reduced severity and frequency of seizure attacks in 6 cases; improvement of life quality in 7 cases; no effect in 6 cases	No side effects were found	Wang et al., 2005
Chronic epilepsy	RCT	Gastrodin (100 mg, *p.o*., tid) plus CBZ (100 mg, *p.o*., tid)/ CBZ (100 mg, *p.o*., tid)	4 weeks	38/36	The effective rate of the treatment group (84.21%) was significantly higher than that of the control group (63.89%); EEG improvement rate of the treatment group (73.33%) was significantly higher than that of the control group (48.00%)	Not mentioned	Lu and Xu, 2015
PD-MCI	RCT	Gastrodin (600 mg, *i.v*., qd) plus madopar/ madopar	12 weeks	35/35	Cognitive improvement in treatment group was significantly higher than that in the control group	One patient had mild side effects of rubefaction, dry nose and dry mouth	Feng et al., 2011
PSD	RCT	Gastrodin (500 mg, *i.v*., qd for 2 weeks; 500 mg, *p.o*., qd for 2 weeks) plus mirtazapine (15–30 mg, *p.o*., qd) / mirtazapine (15–30 mg, *p.o*., qd)	4 weeks	29/29	The effective rate of the treatment group was significantly higher than that of the control group	Not mentioned	Hu and Ren, 2013
VD	RCT	Gastrodin (400 mg, *i.v*., qd)/ piracetam (800 mg, *i.v*., qd)	30 days	40/40	The MMSE score was significantly improved in the treatment group, while no significant improvement was found in the control group did not show significant improvement	Not mentioned	Zhang et al., 2009
VD	RCT	Gastrodin (100 mg, *p.o*., qd) plus donepezil (5 mg, *p.o*., qd) plus / donepezil (5 mg, *p.o*., qd)	12 months	24/24	The improvement of the MMSE, ADL and Hachinski ischemia scales and EEG performance were significantly higher in the treatment group than that in the control group	Not mentioned	He and Wang, 2014
Cognitive decline after mitral valve replacement surgery under anesthesia	RCT	Gastrodin (40 mg/kg, *i.v*., qd) after the induction of anesthesia/saline	–	100/100	The incidence rate of cognitive decline in treatment group is significantly lower than that in control group at discharge (9 vs. 42%) and 3 months later (6% and 31%)	Not mentioned	Zhang et al., 2011
Cognitive decline after radical operation of cervical carcinoma under epidural anesthesia	RCT	Gastrodin (400 mg, *i.v*., qd) after the surgery plus basic treatment / basic treatment	1 week	39/39	Cognitive dysfunction was observed in both groups after the surgery, and compared with the control group, the MMSE score of the experimental group was higher; the levels of CD4+, CD8+ and CD4+/CD8+ were also significantly higher, and serum S100β protein concentration was lower in the treatment group	Not mentioned	Zheng et al., 2016

a PD-MCI, Parkinson's disease combined with mild cognitive impairment; PSD, post-stroke disease; VD, vascular dementia.

b *RCT, randomized controlled trial*.

c *AEDs, anti-epileptic drugs; CBZ, carbamazepine*.

d *EEG, electroencephalogram; MMSE, mini mental state examination; ADL, ability of daily living*.

## *G. elata, rhizoma Gastrodiae*, and gastrodin

*G. elata* (Orchidaceae) is a famous traditional Chinese herb that has been used for centuries. *Rhizoma Gastrodiae*, the dried rhizome of *G. elata*, is the main medicinal part of the plant. Gastrodin is the main bioactive component of *Rhizoma Gastrodiae*.

### Botanical aspects of *G. elata*

*G. elata*, commonly known as *Tianma*, is a saprophytic perennial herb that belongs to the genus *Gastrodia*, family Orchidaceae. It is primarily found in eastern Asia, specifically in the mountainous areas of China, Korea, Japan, and India (Shuan and Chen, 1983; Jones, 1991). In China, it grows mainly in *Sichuan, Guizhou, Yunnan*, and other provinces (Chinese Academy of Sciences, 2004). In ancient times, it was also called *Chijian, Guiduyou, Duyaozhi*, or *Mingtianma*, which are out of use nowadays. It grows in wet place with a lot of humus, especially in humid mountainous areas at an altitude of 400–3,200 m above sea level and depends on the fungus *Armillaria mellea* for nutriments (Wang M.-W. et al., 2007). Great demand has led to overexploitation of the plant, and its wild species has been listed as an endangered species in China (Chen et al., 2011). For this reason, great efforts have been made toward its preservation and cultivation (Chen Y. Y. et al., 2014), and since it was successfully cultivated in the 1970s, home-grown species has gradually become the main source of the plant (Xu and Guo, 2000).

**Figure 3:**
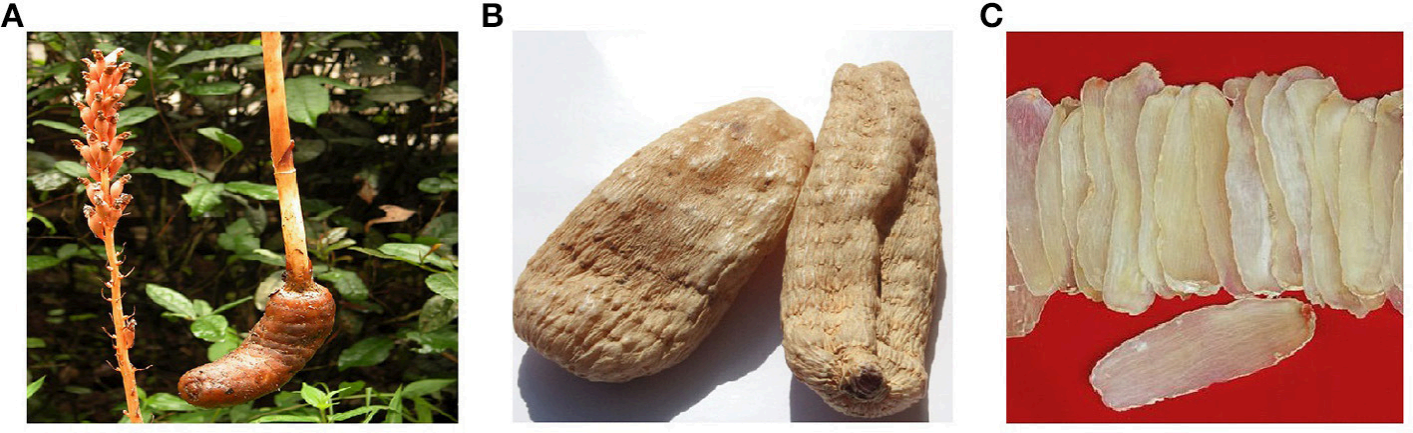
*Gastrodia elata* Blume **(A)** whole plant; **(B)** dried rhizomes; **(C)** decoction pieces.

*G. elata* lives underground during its life cycle except for florescence. The whole plant is free of chlorophyll, which is the unique botanical character of it. It grows to the height of 30–150 cm and the stems are vertical, cylindrical and yellowish red. Its inflorescence is fringy raceme, golden colored and 10–30 cm long. The leaves are scalelike, membranous, nervulose, and about 1–2 cm long. The rhizomes are pachyntic, oblong, about 10 cm long, and 3–4.5 cm in diameter (Chinese Academy of Sciences, 2004; as shown in Figure 3).

### *Rhizoma Gastrodiae* and its traditional use

*Rhizoma Gastrodiae* is the dried rhizome of *G. elata*. Other common names of it include *Tianma, G. elata, Gastrodia* Root, *Gastrodia* Rhizome, *Gastrodia* tuber, and the like. Generally, it is harvested during winter and spring. Those excavated in winter, termed *Gastrodia hiemalis* T. P Lin (*Dong Ma*), are considered to be of superior quality, while those excavated in spring are named *Gastrodia fontinalis* T. P Lin (*Chun Ma*) and of inferior quality (Lei et al., 2015). Once dug out, they are immediately washed clean, braised well and dried at low temperature for clinical use (Chinese Pharmacopoeia Commission, 2015). The dried rhizomes are oval or long strip in shape, about 6–10 cm long and 2–5 cm in diameter. The surface is yellowish-white or yellowish-brown. The texture is hard, keratoid and not easy to break (Chinese Pharmacopoeia Commission, 2015). It is used sliced medicinally (as shown in Figure 3).

The earliest records about the medicinal effects of *Rhizoma Gastrodiae* date back to 2,000 years ago. In *Shennong's Classic of Materia Medica*, the first herbal monograph in Chinese history written in the *Han* Dynasty, *Rhizoma Gastrodiae* was described as a “top grade medicine” that can rejuvenate body, enhance health and extend life without toxicity and can be long-term used without harm (Gu and Yang, 2013). In ancient times, *Rhizoma Gastrodiae* was extensively used for the treatment of headache, dizziness, spasm, epilepsy, infantile convulsion, arthralgia, numbness of the limbs, stoke, amnesia, and other disorders in China. It is also included in the *China pharmacopeia* at present (Chinese Pharmacopoeia Commission, 2015). In recent decades, a large number of experiments have been conducted on its pharmacological properties, and the results showed that *Rhizoma Gastrodiae* have various therapeutic effects including neuroprotective, anti-inflammatory, antioxidative, antiepileptic, anticonvulsive, antipsychotic, anxiolytic, antidepressant, circulatory system modulating, memory-improving effects, etc. (Zhan et al., 2016).

### Gastrodin: sources, pharmacokinetics, and toxicity

#### Sources of gastrodin

Gastrodin is usually obtained by plant extraction or by chemical synthesis. Direct extraction from *G. elata* is the traditional method to obtain gastrodin. In 1978, Zhou et al. (1979) and Feng et al. (1979) isolated gastrodin from the ethanolic extract of *Rhizoma Gastrodiae* for the first time. In the following years, more extracting methods have been developed, such as backflow extraction with water (Teo et al., 2008; Xu et al., 2012), ethanol or methanol (Li and Chen, 2004; Ong et al., 2007), ultrasound-assisted extraction and microwave-assisted extraction (Yu et al., 2010). However, considering that gastrodin content is low in the plant, averaging 0.41% in dried rhizome, this method is relatively expensive and leading to the waste of natural resources (Zhou et al., 1980). Besides, separation and purification procedures are usually complicated. Chemical synthesis is another common way to produce gastrodin. In 1980, Zhou et al. (1980) reported the synthesis of gastrodin using 2, 3, 4, 6-Tetra-O-acetyl-α*-*D-glucopyranosyl bromide as a precursor and the total yield from glucose was 24.00%. However, the use of toxic phenols, phosphate and bromide in this process would cause serious environmental pollution. Although this method was later refined to increase yield and reduce pollution, the use of nonrenewable petroleum was still a problem to the environment (Pang and Zhong, 1984; Dai et al., 2004).

Because of the disadvantages of the two approaches aforementioned, new methods for the production of gastrodin have been under development over the years, and biosynthesis method has been developed in this context. It was reported that exogenous *p*-hydroxybenzaldehyde or *p*-hydroxybenzyl alcohol (HBA) could be transformed to gastrodin by using cell suspension culture of *Datura tatula* L. (Gong et al., 2006; Peng et al., 2008), and a strain of *Rhizopus chinensis* Staito AS3.1165 was also capable of biotransforming *p*-hydroxybenzaldehyde into gastrodin (Zhu et al., 2010). Fan et al. (2013) reported to produce gatrodin from HBA through biotransformation by cultured cells of *Aspergillus foetidus* and *Penicillium cyclopium*. Recently, a more efficient route of microbial synthesis of gastrodin in *Escherichia coli* was reported by Bai et al. (2016). Using a carboxylic acid reductase from *Nocardia iowensis*, a *Rhodiola* UGT73B6 and endogenous alcohol dehydrogenases of *E. coli*, 4-hydroxybenzoic acid, which is a key intermediate derived from chorismate, could be catalyzed to transform to gastrodin. The final recombinant strain produced 545 mg/L gastrodin from glucose in shake flasks in 48 h.

#### Pharmacokinetics of gastrodin

##### General pharmacokinetic characteristics of gastrodin

Gastrodin has been investigated on its pharmacokinetics in various animal models including rats, rabbits and dogs as well as in human (as shown in Table 4). Although there's distinct species specificity among different species, and the administration methods, analytical methods and data interpretations are different, some general conclusions could be reached.

**Table 4:** Pharmacokinetic parameters of gastrodin.

**Species**	**Route^a^**	**Dose**	***C*_max_ or *C*_t_ (μg/mL)^b^**	***t*_1/2β_ (h)^c^**	**AUC_0−∞_ (mg/mL/min)^d^**	**Bioavailability (%)**	**Method^e^**	**References**
Rabbit	*i.v*.	100 mg/kg	437.89 (*C*_5min_)	–	24.35	–	UV	Wang and Wang, 1985
	*p.o*.	100 mg/kg	37.48 (@*t*_max_ 1.08 h)	–	4.15	16.80		
	*i.m*.	100 mg/kg	267.31 (@*t*_max_ 0.58 h)		24.46	102.71		
Rabbit	*i.v*.	100 mg/kg	–	0.64	14.36	–	HPLC	Liu et al., 1986
		200 mg/kg	–	0.79	33.4	–		
	*i.d*.	200 mg/kg	14.4 (@*t*_max_ 0.20 h)	0.73	0.949	2.8		
Rat	*i.v*.	100 mg/kg	–	0.14	3.2	–	–	
		200 mg/kg	–	0.20	7.9	–		
	*i.g*.	200 mg/kg	82.9 (@*t*_max_ 0.50 h)	0.22	7.160	81.0		
Dog	*i.v*.	50 mg/kg	–	1.75	38	–		
Rat	*p.o*.	200 mg/kg	–	–	–	79.9	HPLC	Liu et al., 1988
Human	*i.v*.	600 mg	94.66 (@*t*_max_ 1.0 h)	4.16	0.006517	–	HPLC	Luo et al., 2006
Rat	*i v*.	100 mg/kg	533 (*C*_15min_)	0.65	8.061	–	HPLC	Lin et al., 2007
		300 mg/kg	1033 (*C*_15min_)	0.64	29.882	–		
Rat	*i.d*.	200 mg/kg	84.7 (@*t*_max_ 0.93 h)	0.93	10.466	55.0	HPLC	Wang Q. et al., 2007
	*i.v*.	200 mg/kg	350.9 (*C*_0_)	0.69	19.020	–		
Rat	*i.v*.	50 mg/kg	24.1 (@*t*_max_ 0.25 h)	–	–	–	LC-MS/MS	Lin et al., 2008
Human	*p.o*.	200 mg	1.48455 (@*t*_max_ 0.81 h)	6.06	0.007210	–	HPLC-PDA	Ju et al., 2010
Rat	*i.v*.	40 mg/kg	75.6 (*C*_0_)	1.14	1.338	–	HPLC	Jiang et al., 2013
	*i.g*.	40 mg/kg	21.7 (@*t*_max_ 0.250 h)	2.81	1.086	81.2		
Dog	*i.g*.	40 mg/kg	23.00 (@*t*_max_ 1.83 h)	4.80	7.305	–	UFLC-ESI-MS/MS	Jia et al., 2014
Rat	*i.g*.	40 mg/kg	21.74 (@*t*_max_ 0.25 h)	2.81	1.088	–	HPLC	Jiang et al., 2015
	*i.v*.	20 mg/kg	75.65 (*C*_0_)	1.14	1.336	–		
Rat	*i.g*.	100 mg/kg	44.84 (@*t*_max_ 0.42 h)	1.13	3.475	–	UHPLC-FLD	Tang et al., 2015
Rat	*i.g*	21 mg/kg	10.78 (@*t*_max_ 0.38 h)	1.43	0.567	33.9	HPLC	Liu et al., 2015
	*i.v*.	21 mg/kg	–	1.30	1.684	–		

a *i.v., intravenous administration; i.p., intraperitoneal administration; i.m., intramuscular administration; p.o., oral administration; i.d., intraduodenal administration*.

b C_max_, maximal/peak plasma concentration; t_max_, time to reach peak plasma concentration.

c *t_1/2β_, elimination half-life*.

d *AUC, area under the curve*.

e *UV, ultroviolet spectrometry method; HPLC, high performance liquid chromatography; LC-MS/MS, liquid chromatography technique coupled to tandem mass spectrometry; HPLC-PDA, HPLC method coupled with photodiode array detector; UFLC-ESI–MS/MS, ultra fast liquid chromatography-electrospray ionization-tandem mass spectrometry; UHPLC-FLD, ultra high performance liquid chromatography-fluorescence detection*.

First, the absorption of gastrodin in the intestine is fast. In rats, gastrodin was detectable in plasma in < 5 min after intragastric (*i.g*.) administration (100 mg/kg) and the time to reach the maximum plasma concentration (*t*_max_) was only 0.42 h (Tang et al., 2015). In human, after a gastrodin capsule (200 mg) was orally administered, gastrodin was quickly absorbed with an absorption half-life of 0.18 h and a *t*_max_ of 0.81 h (Ju et al., 2010). The oral bioavailability of gastrodin varies greatly among different species. In rats, the oral bioavailability was reported to be as high as around 80% (Liu et al., 1986, 1988; Jiang et al., 2013), while in rabbits it was only 16.80% (Wang and Wang, 1985). No data on the bioavailability of gastrodin in human has been reported.

Studies using Caco-2, MDCK and Bcap37 cells as *in vitro* models suggest that the transport of gastrodin is mainly by passive paracellular transport pathway (Wang Q. et al., 2007; Wang and Zeng, 2010). Glucose transporters (GLTs) were reported to be involved in the intestinal absorption of gastrodin, thus glucose and GLT inhibitors could affect the intestinal absorption process (Cai et al., 2013). When gastrodin was orally co-administered with four times higher dose of glucose, the *t*_max_ was prolonged from 28 to 72 min in rats, while the *t*_max_ was significantly shortened in diabetic rats because of the high intestinal GLT level (Cai et al., 2013). Gastrodin is not a substrate or inhibitor of P-glycoprotein (P-gp), therefore P-gp did not participate in the absorption of gastrodin in the intestine or in the transport across the blood-brain barrier (BBB) (Wang Q. et al., 2007; Wang and Zeng, 2010).

Second, gastrodin is distributed rapidly and widely after entering the systematic circulation. The drug-protein binding ratio of gastrodin in rat plasma is low (<8%) (Lu et al., 1985; Liu et al., 1988), which might be due to gastrodin's high hydrophilicity, thus gastrodin usually occurs in free form in plasma (Lu et al., 1985). In rats, it was detectable in kidney, liver, lung and spleen in 2 min after intravenous (*i.v*.) administration, and the time to reach maximum concentration in kidney, liver, bile, and brain were <15 min (You et al., 1994; Lin et al., 2007, 2008; Wang et al., 2008), indicating a rapid distribution. Gastrodin is mainly distributed to kidney, lung, liver, spleen, gastrointestinal tract, and brain in rats (Lu et al., 1985; Liu et al., 1988, 2015; You et al., 1994), while it is hardly distributed to muscle and fat (Lu et al., 1985). The existence of an enterohepatic circulation of gastrodin was also observed (You et al., 1994; Wang Q. et al., 2007).

Third, the elimination of gastrodin is also quick. The half-lives (*t*_1/2_) of gastrodin in rat and human plasma were reported to be short. Liu et al. (2015) reported that in rats, the *t*_1/2_ in plasma was 1.13 and 1.30 h when *i.g*. (21 mg/kg) and *i.v*. (21 mg/kg) administered, respectively. Ju et al. (2010) reported that in human plasma, the distribution half-life of gastrodin was 3.78 h and the elimination half-life was 6.06 h. HBA is the main metabolite of gastrodin. After oral administration, gastrodin was quickly transformed into HBA in intestines, plasma, kidney, liver and brain (You et al., 1994; Wang Q. et al., 2007). Lin et al. (2008) reported that HBA was found in the bile and brain in 10 min and reached peak concentration in bile and brain at 15 min after *i.v*. administration of gastrodin (50 mg/kg) in rats, and its levels also declined rapidly. The majority of gastrodin is excreted unchanged in the urine, which is probably due to its low molecular weight < 300, and a small proportion of gastrodin undergoes biliary excretion (Lin et al., 2007). HBA is excreted mainly through hepatobiliary system (Lin et al., 2007). These results indicated a short action time of gastrodin in the body, which might limit its clinical therapeutic. Thus, multi-dose administration was required to prolong the action time.

##### Brain pharmacokinetics of gastrodin

Considering that the main therapeutic effects of gastrodin are exerted in CNS, the brain pharmacokinetics of gastrodin has drawn much attention. Gastrodin is able to pass though the BBB and distribute in the brain quickly after it enters the systematic circulation (Lin et al., 2007, 2008; Liu et al., 2015). Lin et al. (2008) reported that gastrodin could be detected in brain in 5 min after *i.v*. administration (50 mg/kg) and reach peak brain concentration in 15 min in rats. But the extent of brain exposure of gastrodin was rather small, with a brain-to-blood distribution ratio of only 0.007 at the dose of 100 mg/kg (*i.v*.) in rats (Lin et al., 2007), which might be due to its rapid metabolism into HBA as well as its poor BBB permeability. However, the concentration of HBA is relatively high in the brain. You et al. (1994) reported that HBA accounted for 42.3% of the total radioactivity of gastrodin and HBA in rat's brain 0.5 min after *i.v*. administration. There are two sources of brain HBA: the major source is plasma HBA, for HBA is a lipophilic compound with high BBB permeability, thus the plasma HBA is easy to get into the brain, and the minor source is those metabolized by brain gastrodin (You et al., 1994; Wang et al., 2008). The brain concentration of HBA also declines rapidly (You et al., 1994; Jia et al., 2014).

There have been debating opinions upon the role played by gastrodin and HBA in gastrodin's neuropharmacological effects, for HBA could also exert pharmacological effects on CNS by itself by binding to benzodiazepine receptor in brain membrane (Guo et al., 1991). Some early reports suggested that the HBA passing through BBB was the main active compound that exerted pharmacological effects (Lu et al., 1985; You et al., 1994), but recent studies suggested that gastrodin might work by itself in the brain (Wang et al., 2008; Liu et al., 2015). However, both gastrodin and HBA are not sufficient to explain gastrodin's wide range of CNS effects considering gastrodin's poor BBB permeability, HBA's limited biological effects, and short action time of both compounds. There might be some possible explanations for this as follows. First, the dosage of gastrodin is always high in clinical use as well as in preclinical models, for gastrodin has been proven to be a non-toxic compound in acute and subacute toxicity experiments, thus a certain amount of gastrodin could enter CNS despite of its poor BBB permeability. Second, some suggest that gastrodin might have a relatively strong potency thus the small amount of free gastrodin in the brain may cause significant pharmacological effect in CNS (Lin et al., 2007). Third, HBA might have other biological actions on CNS for the studies on HBA's pharmacological properties are still limited. Fourth, there might be other metabolites of gastrodin in the brain. When gastrodin was *i.v*. administered (50 mg/kg) to rats, Lin et al. (2008) observed an unknown peak in brain dialysate using liquid chromatography technique which displayed an identical fragment pattern with HBA, indicating it might be an isomer of HBA. The unknown substances might also have CNS effect. Nevertheless, more studies are still in need to better elucidate the bioactive component that reaches the brain as well as the roles of gastrodin and its metabolites in the claimed neuropharmacological effects.

#### Toxicity and safety of gastrodin

Acute toxicity experiments suggest that gastrodin and its metabolite HBA are safe on acute administration. In mice, oral administration of gastrodin or HBA at the dosage up to 5000 mg/kg caused no mortality or apparent toxic effects. Therefore, no assay could be made for median lethal dose in mice (Deng and Mo, 1979). In rabbits, when orally administered of gastrodin at the dosage of 6,000 mg/kg, no toxic effects or mortality were observed, either (Chai et al., 1983). Intravenous administration of gastrodin (5 mg/kg) caused no changes in heart rhythm in rabbits, but slightly reduced the heart rate, which would return to normal in 2 h (Mo and Deng, 1980). These results indicated a high margin of safety in acute toxicity studies. Subacute toxicity studies in dogs and mice also signified a safety profile of gastrodin. When gastrodin or HBA was orally administered at the dosage of 75 mg/kg/d for 14 d in dogs, or at the dosage of 375 mg/kg/d for 60 d in mice, the appetite and activities of the animals were unchanged, and no abnormal histological findings in heart, lung, liver, spleen, kidney, stomach, and intestine were found. In addition, no change in the blood counts, glutamic-pyruvic transaminase, cholesterol, or non-protein nitrogen in the blood were observed, either (Mo and Deng, 1980).

Although the toxicity studies in animals revealed that gastrodin is relatively safe to use, cases of clinical adverse drug reaction (ADR) or event (ADE) induced by gastrodin were reported occasionally (Zheng et al., 2015). In a retrospective study including 315 cases with ADR or ADE induced by gastrodin in Chongqing province of China from January 2008 to June 2014, the ADR or ADE were mainly happened in gastrointestinal system, skin, and nervous system. The most frequently reported symptoms included rash, pruritus, dizziness, dry mouth, nausea, palpitation, vomiting, and headache (Zheng et al., 2015). There is still a need for carrying out more detailed toxicity study of gastrodin according to the International Council for Harmonization safety guidelines.

## Effects of gastrodin on CNS diseases

### Epilepsy

Epilepsy is a group of chronic disorders of the brain characterized by recurrent episodes of seizures due to abnormal excessive electrical discharges of cerebral neurons (Chang and Lowenstein, 2003). Although there is an increase in the number of available antiepileptic drugs (AEDs), the cure rate of the disease has not been improved substantially, calling for a need to identify new drugs that could treat or protect against epilepsy. It was demonstrated that pretreatment of gastrodin could significantly prolong the seizure latency (Deng and Mo, 1979) as well as reduce seizure severity, shorten seizure duration, accelerate recovery and decrease mortality rate *in vivo* (Chai et al., 1983). Later, the mechanisms underlying the anticonvulsant effect of gastrodin were also explored.

Imbalance between the activities of inhibitory neurotransmitters and excitatory neurotransmitters is considered to be the key mechanism associated with the abnormality of neural activities. Gamma-aminobutyric acid (GABA) is the major inhibitory neurotransmitter in the brain (During et al., 1995) and enhancement of the activity of GABA would be useful to treat epilepsy. Baek et al. (1999) reported that preincubation of gastrodin could irreversibly inactivate the succinic semialdehyde dehydrogenase (SSADH), a GABA degradative enzyme, of bovine brain *in vitro* in a time-dependent manner. Later, a more comprehensive study *in vivo* evidenced that gastrodin could inhibit the activities of GABA transaminase (GABA-T) and succinic semialdehyde reductase (SSAR) as well as SSADH, all of which are enzymes responsible for GABA degradation, in the hippocampus of seizure-sensitive gerbils (An et al., 2003). These results suggested that gastrodin could reverse the decrease of GABA level in the synaptic cleft by inhibiting its degradation. However, it was reported that GAD65 and GAD67, two GABA-synthetic enzymes, were not associated with the anti-convulsive effect of gastrodin (An et al., 2003). Apart from enhancing GABA activity, gastrodin was also discovered to decrease the activity of glutamate (Glu), which is the most important excitatory neurotransmitter in the brain. Chen and Tian (2009) found that gastrodin could reduce the number of Glu immunohistochemically positive cells in the hippocampus of a pentetrazol (PTZ)-induced rat model. In another study, Mu et al. (2009) reported gastrodin was able to inhibit the expression of matabotropic glutamate receptor 1 (mGluR1), a type of Glu receptor that are active through an indirect metabotropic process, to reduce the activity of Glu. However, gastrodin was found not to be interacting with N-Methyl-D-Aspartate (NMDA) receptor, an ionotropic Glu receptor, to inhibit NMDA receptor-facilitated seizures. In an *in vitro* model using hippocampal slices cultures, gastrodin failed to inhibit epileptiform discharges induced by Mg^2+^-free medium, which is believed to be NMDA receptor-mediated spontaneous activity. In the same study, they also established an *in vivo* model by treating rats with lithium-pilocarpine (Li-pilo), which was facilitated with NMDA receptor activation, to induce generalized tonic-clonic seizures. Gastrodin was unable to prevent seizure attacks, reduce seizure duration, decrease death rate, or alleviate neuronal loss, suggesting gastrodin has no effect on NMDA receptor-mediated seizures (Wong et al., 2016).

It has been established that brain inflammation plays an important role in epileptogenesis (Vezzani et al., 2011), thus regulating the associated factors might be a therapeutic strategy for epilepsy. Chen et al. (2017) demonstrated that gastrodin was able to inhibit the increase of interleukin-1beta (IL-1β) and tumor necrosis factor-alpha (TNF-α) levels, both of which were pro-inflammatory cytokines, as well as reverse the decrease of interleukin-10 (IL-10) level, an anti-inflammatory cytokine in the brain of PTZ-induced mice. In the meantime, gastrodin could inhibit the expression of mitogen-activated protein kinases (MAPK), cAMP response element binding protein (CREB), and NF-κB, suggesting that gastrodin attenuates seizures by inhibiting the MAPK-associated inflammatory responses (Chen et al., 2017). Anti-oxidative effect was also found to be involved in the anti-epileptic and neuroprotective effect of gastrodin (Li and Cheng, 2015; Zhong et al., 2016). In a Li-pilo-induced rat model, pretreatment of gastrodin could inhibit the decrease of catalase (CAT), glutathione (GSH), superoxide dismutase (SOD), glutathion reductase (GR) and total antioxidation (T-AOC) and alleviate the increase of malondialdehyde (MDA) level, reported by Zhong et al. (2016). In addition, gastrodin could also inhibit apoptosis by increasing the Bcl-2 expression and decreasing the Caspase-3 expression in the brain of a Li-pilo-induced rat model (Bian et al., 2016).

Aberrant neurogenesis contributes to the development of epilepsy. In a rat model of PTZ-kindling epilepsy, Lian et al. (2005) discovered that GAP-43, an intrinsic presynaptic determinant for neurite outgrowth and plasticity, was overexpressed in model rats, while pretreatment of gastrodin could decrease the expression of GAP-43 as well as reduce the susceptibility of epileptic seizures and decrease the seizure score. Connexin 43, a protein involved in the formation of abnormal gap junction, and synaptophysin, which was associated with the formation of synaptic reconstruction, were also found overexpressed in PTZ-kindling rats, while these process could be inhibited by pretreatment of gastrodin, reported by Cao et al. (2008a,b).

It is surprising that few clinical studies have been conducted evaluating the anti-epileptic effect of gastrodin despite the extensive reports in preclinical models. In 2005, Wang et al. (2005) investigated the therapeutic effect of gastrodin as an adjuvant therapy for the treatment of refractory epilepsy. In this study, 15 patients of refractory epilepsy received gastrodin tablets (300 mg/kg) on the basis of the original AEDs. As a result, the severity and frequency of seizure attacks were significantly reduced. In seven cases, patients reported improvement of life quality and reduce of other symptoms including trouble sleeping, limb numbness, or fatigue. There was no effect in six cases. In a randomized controlled trial (RCT) evaluating the effect of gastrodin on epilepsy (Lu and Xu, 2015), 74 patients were randomized to treatment group (38 cases), receiving gastrodin (300 mg, *p.o.*, tid) combined with Carbamazepine (CBZ) (100 mg, *p.o.*, tid), or control group (36 cases), receiving CBZ only. After 4 weeks, the effective rate of the treatment group (84.21%) was significantly higher than that of the control group (63.89%) (*P* < 0.05). Consistent with this result, the treatment group had higher EEG improvement rate (73.33%) than the control group (48.00%) (*P* < 0.05). These data suggest that gastrodin has potential benificial effect on epilepsy as an adjuvant therapy. However, the sample populations of the above studies were small, and the methodological qualities were relatively low. Large-scale, multicenter, long-term, placebo-controlled RCTs are still in need to provide more solid clinical evidence.

### Neurodegenerative disease

#### Alzheimer's disease (AD)

AD is the leading cause of dementia worldwide, accounting for more than 60% of total cases. Its pathological changes are characterized by extracellular senile plaques, intracellular neurofibrillary tangles, and extensive neuron loss, but the underlying mechanism remains unclear, and no curative treatments for AD are available (Scheltens et al., 2016). *In vivo* and *in vitro* studies have shown that gastrodin has potential therapeutic effects for treating AD. Liu et al. (2005) established a cellular model of AD with primary cultured cerebral cortical and hippocampal cells induced by Aβ_25*-*35_, and found that gastrodin could significantly increase cell viability and decrease lactic dehydrogenase (LDH) release, indicating that gastrodin could protect neurons from Aβ-induced damage. Liu and Wang (2012) found that gastrodin could improve learning/memory ability of an AD mouse model after 4 weeks of treatment. However, the potential mechanisms under the therapeutic effects remain unclear.

Amyloid beta (Aβ) peptide, the main component of senile plaques, is regarded as the pivotal toxicant of AD. It is derived from the amyloid precursor protein (APP) which is cleaved by β and γ-secretases in the amyloidogenic pathway. Zhu et al. (2014) reported that gastrodin could dose-dependently reduce the levels of extracellular and intracellular Aβ *in vitro*, and further studies (Zhou et al., 2016) suggested the inhibiting mechanisms were related to the reduction of β and γ-secretase activities. Zhang et al. (2016) found gastrodin could suppress β-secretase expression by inhibiting the protein kinase/eukaryotic initiation factor-2alpha (PKR/eIF2alpha) pathway in an AD mouse model. However, IDE, a zinc metalloprotease known to degrade Aβ, was reported to be not associated with the inhibition of Aβ aggregation induced by gastrodin (Liu et al., 2011).

Aβ could elicit a variety of neuropathological changes such as oxidative stress, inflammatory responses, altered neuronal activity, synaptic loss, dysfunction of neuronal transmission, and neuronal death (Dawkins and Small, 2014). Studies have shown that gastrodin could protect cells from Aβ-induced neurotoxicity. Zhao et al. (2012) reported gastrodin was able to improve cell viability by improving the activity of SOD and CAT in an Aβ_1*-*42_-induced mouse model of AD, and this anti-oxidative effect was correlated with the upregulation of the expression of ERK1/2-Nrf pathway. Aβ could activate glial cells to produce several inflammatory factors, while Hu et al. (2014) reported that gastrodin could alleviate the activation of microglial cells and astrocytes in a mouse model of AD. He also found a decrease of the overexpression of TNF-α and IL-1β in lipopolysaccharide (LPS)-stimulated N9 cells when pretreated with gastrodin, which confirmed the anti-inflammatory effects of gastrodin. Endoplasmic reticulum (ER) stress signaling was also involved in the Aβ-induced microglial activation and apoptosis. Lee et al. (2012) found that the anti-apoptotic ER stress protein glucose-regulated protein 78 (GRP78), a marker for enhanced protein folding machinery, was decreased in Aβ-stimulated microglial cells, while pretreatment of gastrodin increased the expression of GRP78, revealing the protective effects of gastrodin was possibly achieved through the enhancement of protein folding machinery, thus attenuating the neurotoxic activities of microglial cells. Li and Qian (2016) found gastrodin could not only counteract Aβ_1*-*42_-triggered release of pro-inflammatory cytokines and nitric oxide (NO), but also attenuate Aβ_1*-*42_-induced apoptosis in primary neural progenitor cells (NPCs), and these effects were associated with the inhibition of MEK1/2 and JNK expressions. Chen P. Z. et al. (2014) discovered that gastrodin could inhibit Aβ_1*-*42_-induced aberrant spontaneous discharge in the entorhinal cortex (EC) of rats in a concentration-dependent manner and the effect might be partially mediated by its inhibitory action on Aβ-elicited inward currents in EC neurons. Brain-derived neurotrophic factor (BDNF) is an important neurotrophin in the brain that has specific and dose-response protective effects on neuronal toxicity (Arancibia et al., 2008). In a tree shrew AD model, the inhibition of BDNF expression was reversed by gastrodin after 30 days of treatment (He et al., 2013).

In summary, *in vivo* and *in vitro* studies demonstrated that gastrodin could inhibit the production and aggregation of Aβ, as well as protect neurons against Aβ-induced injury. However, no clinical data describing the effects of gastrodin on human AD has been published. Considering the fact that seizures are a common symptom of AD and the anti-convulsant effects of gastrodin described previously, further investigation of therapeutic potential of gastrodin on human AD is merited.

#### Parkinson's disease (PD)

PD is primarily a movement disorder characterized by rest tremor, bradykinesia, rigidity, and loss of postural reflexes (Jankovic, 2008). The patients may also suffer from functional disability, reduced quality of life, and rapid cognitive decline. The death of dopaminergic neurons in the substantia nigra that results in a loss of tyrosine hydroxylase (TH) positive neurons and reduced dopamine (DA) levels in the striatum is responsible for the motor symptoms. Recent studies suggest that gastrodin is also protective against PD pathological changes. In a PD rat model established by injecting 6-hydroxydopamine (6-OHDA) to the right midbrain ventral tegmental area (VTA), gastrodin could improve rotation behavior of PD rats and increase the expression of TH-positive neurons in VTA, showing a protective effect on TH-positive neurons (Wang and Huang, 2013). Gastrodin could also reduce apoptosis *in vivo* and *in vitro* (Kumar et al., 2013; Xi and Ren, 2016). In a 6-OHDA-induced rat model, gastrodin could inhibit the decrease of Bcl-2 mRNA and increase of Bax mRNA (Xi and Ren, 2016). In 1-methyl-4-phenylpyridinium (MPP^+^)-stimulated SH-SY5Y cells, pretreatment of gastrodin was able to inhibit apoptosis by regulating free radicals, Bax/Bcl-2 mRNA, caspase-3, and cleaved poly ADP-ribose polymerase (PARP) (Kumar et al., 2013).

Wang X. L. et al. (2014) treated C57BL/6 mice with 1-methyl-4-phenyl-1,2,3,6-tetrahydropyridine (MPTP), which could induce mitochondrial dysfunction and thereby cause PD-like behavioral symptoms, to establish a PD model. They found that treatment of gastrodin could prevent MPTP-induced oxidative stress as measured by MDA in midbrain. Furthermore, it was also able to significantly increase HO-1, SOD, GSH levels as well as ERK1/2 phosphorylation and Nrf2 nuclear translocation in striatum, suggesting that gastrodin protect midbrain of MPTP-intoxicated mice against oxidative stress through upregulating ERK1/2-Nrf2 pathway.

Glial cell activation also plays an important role in the pathology of PD. Microglial cells mediate immune responses by secreting many factors such as cytokines, chemokines, prostaglandins, ROS, RNS, and growth factors, some of which could enhance oxidative stress and trigger apoptotic cascades in neurons (Tansey and Goldberg, 2010). Li et al. (2012) reported that gastrodin was able to reduce the number of activated microglial cells and down-regulate nigral IL-1β and TNF-α expression in a rotenone-induced PD rat model, indicating gastrodin could alleviate microgial cells activation in PD substantia nigra. Astrocytes are also an important component in the pathology of PD (Finsterwald et al., 2015). In a MPTP-induced mouse PD model, gastrodin reduced reactive astrogliosis and prevented DA depletion as assessed by immunohistochemistry and immunoblotting in the substantia nigra and striatum of mice (Kumar et al., 2013). Gap junction connexin 43 (Cx43) are coupled with astrocytes and involved in the release of ATP and Glu. Wang and Huang (2013) reported that the expression of Cx43 and the quantity of gap junction intercellular communication (GJIC) was increased in a rat model of PD induced by rotenone, and treatment of gastrodin significantly inhibit this activity, suggesting gastrodin might prevent PD by reducing the expression of Cx43.

Clinical data investigating the therapeutic effect of gastrodin on PD are still limited. In a RCT, the effects of gatrodin on mild cognitive impairment (MCI) of PD patients were evaluated. 70 patients of PD with mild cognitive impairment (PD-MCI) were instructed to orally take gastrodin (600 mg/d) plus madopar or madopar alone for 12 weeks. The outcome demonstrated that cognitive improvement was observed in both groups and the test group performed significantly better than the control group (*P* < 0.01), suggesting that gastrodin might be an effective adjuvant drug for this condition (Feng et al., 2011). However, gastrodin for the motor deficits of PD has not been evaluated and needs further investigation.

### Affective disorders

#### Anxiety

Studies have found that gastrodin could reverse the anxiety-like behavior in the open field test (OFT) and elevated plus-maze test in post-traumatic stress disorder (PTSD) rat model induced by enhanced single prolonged stress (ESPS), demonstrating that gastrodin has anxiolytic effect *in vivo* (Peng et al., 2013). To investigate the underlying mechanism, Peng et al. (2013) discovered that gastrodin could reverse the elevation of IL-6 and IL-1β levels and upregulated expression of iNOS and phospho-p38 MAPK in hippocampus of PTSD rat model, indicating the anxiolytic effect was associated with modulating inflammatory factors and iNOS/p38 cascades.

#### Depression

The anti-depressant effect of gastrodin has been established in many preclinical models as well. Studies have shown that it could reverse the depression-like behaviors in sucrose preference test (SPT), forced swim test (FST), and OFT *in vivo* (Wang H. et al., 2014; Zhang et al., 2014; Chen et al., 2016; Lee et al., 2016; Lin et al., 2016; Sun et al., 2017). The potential mechanisms of the above effect were also investigated.

It has been widely accepted that dysfunction within the monoaminergic neurotransmitter systems play important roles in the pathophysiology of depression, and regulation of the activities of serotonin (5-HT), norepinephrine (NE), and DA might be important targets for the treatment of the disease. Chen et al. (2016) established a FST-induced depression rat model and access the levels of 5-HT and DA as well as their metabolites. 5-HT1A receptor expression and the neuronal cytoskeleton remodeling-related proteins were also evaluated. They found that the monoamine metabolism including 5-HT to 5-HIAA in the hippocampus and DA to DOPAC and HVA ratios was significantly increased and the expression of Slit1 and RhoA, two neuronal cytoskeleton remodeling-related negative regulators, were significantly up-regulated in model rats. However, oral administration of gastrodin could reverse all the changes aforementioned, indicating the anti-depressant effects of gastrodin was associated with decreasing monoamine metabolism and modulating cytoskeleton remodeling-related protein expression in the Slit-Robo pathway. In another study, Lin et al. (2016) also reported that gastrodin could restore the cerebral turnover rates of 5-HT and DA and decrease serum corticosterone levels significantly in unpredictable chronic mild stress (UCMS)-induced rat model as well. Lee et al. (2016) reported that gastrodin could restore single prolonged stress (SPS)-induced NE concentrations increase in hippocampus as well as TH expression in the locus coeruleus. Furthermore, the administration of gastrodin attenuated SPS-induced decreases in the hypothalamic expression of neuropeptide Y and the hippocampal mRNA expression of BDNF.

In a chronic unpredictable stress (CUS)-induced model of rat, Wang H. et al. (2014) found that gastrodin could reverse the overexpression of p-iκB, NF-κB, and IL-1β as well as up-regulate NSCs proliferation in hippocampus of the model. They also discovered that gastrodin did not increase the viability of NSCs alone *in vitro* but could protect them from IL-1β-induced damage. The results suggested that gastrodin may attenuate inflammation and protect hippocampal NSCs from inflammatory injury. Zhang et al. (2014) reported that the expression of glial fibrillary acidic protein (GFAP), a major protein of astrocyte intermediate filaments and a specific marker for astrocytes, and BDNF were both decreased in the hippocampus in a CUS-induced depression rat model, and gastrodin could reverse these changes. They also discovered that gastrodin could improve phospho-ERK1/2 and BDNF levels in hippocampal-derived astrocytes *in vitro*, and although it did not increase the cell viability of astrocytes, it could protect astrocytes from 72 h's serum-free damage. These results indicate that the anti-depressant effect of gastrodin was associated with the improvement of BDNP level and the modulation of astrocytes activation.

The clinical data on the therapeutic potential of gastrodin in affective disorders are scarce. Hu and Ren (2013) evaluated the effect of gastrodin on post-stroke depression (PSD) in a clinical trial. 58 PSD patients were randomized into two groups. Both groups were received mirtazapine (15–30 mg/d) for 4 weeks, and the treatment group used gastrodin (500 mg/d, *i.v*. for 2 weeks, 500 mg/d, *p.o*. for 2 weeks) as an adjuvant therapy. As a result, the effective rate and the decrease of Hamilton Depression Scale (HAMD) score of the treatment group was higher than that of the control group (*P* < 0.05). Although the results indicated gastrodin might be effective for PSD as an adjuvant therapy, more potent evidence is still necessary.

### Cerebral ischemia-reperfusion (I/R) injury

Cerebral I/R refers to cerebral ischemia-induced brain damage that is aggravated by the restoring of the blood supply (Winquist and Kerr, 1997). It often leads to a series of consequences including excitatory amino acid (EAA)-induced neurotoxicity, Ca^2+^ overload, mitochondrial dysfunction, ROS generation, cell apoptosis and inflammatory reaction, which ultimately result in irreversible brain injury. Studies *in vivo* and *in vitro* have demonstrated that gastrodin exhibits neuroprotective effects against cerebral I/R injury. Xue et al. (1998) established an *in vitro* cerebral I/R model with primary cultured cortical neurons by using the method provided by Goldberg et al. (1986). In this study, neurons undergone simulated ischemia were swollen with increased LDH release and lipid peroxide (LPO) content and decreased cell membrane fluidity, while this condition was further aggravated 18 h after the neurons undergone simulated reperfusion. However, when incubated with gastrodin, the neurons were less damaged when they underwent the same two simulated environments, suggesting that gastrodin could protect against the injury caused by I/R. In *in vivo* studies, gastrodin treatment, whether administered before or after the operation, was reported to improve neurological functions, decrease the infarct volume and edema volume in cerebral I/R rat models induced by transient middle cerebral arterial occlusion (MCAO) (Zeng et al., 2006; Bie et al., 2007; Peng et al., 2015), and the neuroprotective effect was observed even 7 days after reperfusion (Peng et al., 2015), suggesting that gastrodin has a long-lasting neuroprotective action.

The mechanisms of gastrodin protecting against I/R were also investigated. EAA-induced neurotoxicity is thought to be the principal pathological mechanism in ischemic brain damage. Under ischemic conditions, the level of extracellular Glu, the most important EEA, is increased markedly, which could stimulate EAA receptors and lead to excessive influx of Ca^2+^ into the neurons and abnormal production of oxygen-derived free radicals, and finally result in neuronal death (Choi, 1988). In an *in vitro* study, Zeng et al. (2006) evaluated the effect of gastrodin on oxygen/glucose deprivation (OGD) or Glu-induced injury in cultured neurons. The results indicated that gastrodin treatment significantly inhibited OGD- and Glu-induced neuronal cell death. Furthermore, they found gastrodin could also alleviate OGD-induced extracellular Glu increase and Ca^2+^ overload. It is also believed that the imbalance between EEAs and inhibitory amino acids (IAAs) contributes to ischemic brain injury (Bogaert et al., 2000). In an *in vivo* study, using a MCAO I/R rat model, Bie et al. (2007) evaluated the effect of gastrodin on four sorts of amino acids including two EEAs, Glu and aspartic acid (Asp), and two IAAs, GABA, and taurine (Tau). They found that ischemia could cause surprisingly increase of the levels of four amino acids in ischemia area and pretreatment of gastrodin before the operation could notably inhibit the rise of the four amino acids levels in the process of ischemia. Furthermore, the ratio of Glu to GABA was increased sharply after ischemia and declined to a certain degree after reperfusion in the model rats, and treatment of gastrodin was able to lower the Glu/GABA ratio (Bie et al., 2007; Zeng et al., 2007). The results indicated that gastrodin could significantly inhibit the release of cerebral amino acids, especially the EEAs, thus modulate the imbalance between EEAs and IAAs in I/R process.

A growing body of research has evidenced that oxidative stress and inflammation are significant contributing factors to the I/R-induced brain injury. During the process of I/R, a burst of ROS is produced, which lead to oxidative stress and initiate the expression of inflammatory factors such as TNF-a, IL-1β, and IL-6 (Olmez and Ozyurt, 2012). Gastrodin was reported to improve anti-oxidant and anti-inflammatory activities in the ischemic areas of the brain *in vivo*. In MCAO I/R rat models, gastrodin was able to reverse the increased content of MDA and enhanced expression of TNF-α, IL-1β (Li et al., 2014; Peng et al., 2015), COX-2 and iNOS (Liu et al., 2016), and increase the SOD activity and expression of HO-1, SOD1 in I/R brain (Peng et al., 2015). These results demonstrated the anti-oxidative and anti-inflammatory effects of gastrodin. Apoptosis is an important step in I/R-induced neuronal death. In an MCAO rat model, gastrodin was able to preserve the expression of anti-apoptotic protein Bcl-2 and suppress expression of pro-apoptotic Bax protein (Peng et al., 2015; Liu et al., 2016) and cleaved caspase 3 (Nie et al., 2012) induced by cerebral I/R. Furthermore, Akt phosphorylation and Nrf2 expression were also significantly increased by gastrodin, indicating the activation of Akt/Nrf2 pathway may play a critical role in the pharmacological action of gastrodin (Peng et al., 2015).

Astrocytes have diverse and important functions in many aspects of ischemic brain damage (Rossi et al., 2007). Under ischemic conditions, the expression of iNOS in astrocytes is elevated, leading to excessive generation of NO, which could cause neurotoxic activities. Hu et al. (2001) reported that the expression of GFAP, the release of LDH and the expression of NOS were all increased in actrocytes under simulated I/R environments *in vitro*, while gastrodin was able to reverse the above changes. The result indicated that gastrodin was able to protect astrocytes against I/R injury and inhibit the expression of NOS and further reduce the NO-induced neurotoxicity.

In summary, the pharmacological actions of gastrodin in protecting brain from I/R-induced injury include alleviating EEA-induced neurotoxicity, exerting anti-oxidative, anti-inflammatory and anti-apoptotic activities and suppressing astrocytes activation.

### Cognitive impairment (CI)

#### Vascular dementia (VD)

VD is a constellation of cognitive and functional impairment associated with cerebrovascular brain injury. It is also the second most prevalent cause of dementia after AD and accounts for around 15% of total cases (O'Brien and Thomas, 2015). For now, there are no proven treatments to stop or reverse the progression of the disease except for some drugs that have only slight symptomatic effects and many adverse reactions. Gastrodin has shown interesting potential for use in the treatment of VD. Zhang L. D. et al. (2008) established a VD model of rat by MCAO and discovered that gastrodin could significantly improve the learning/memory ability of the model, increase the activity of choline acetyltransferase (ChAT) and decrease the activities of acetylcholinesterase (AChE) and Glu, suggesting that the mechanism under the memory-improving effect was related to the upreguation of brain cholinergic system function. Oxidative stress was found to be significantly involved in the pathophysiology of VD. Li and Zhang (2015) established a VD model induced by chronic cerebral ischemia using 2-vessel occlusion (2-VO) method and studied the potential protective effects of gastrodin on cognitive function and tissue oxidative stress of the model. As accessed by Morris water maze, they found that the learning/memory function of the rats was restored by treatment of gastrodin. Furthermore, gastrodin partially decreased the levels of MDA and restored the levels of GSH-Px and total thiol of the model. These results indicated that gastrodin is protective on the learning and memory function of the VD rat model and this effect might be associated with the anti-oxidative effect of gastrodin.

Apart from the preclinical experiments described above, two clinical trials were also conducted to evaluate the therapeutic effect of gastrodin on VD patients in China. Zhang et al. (2009) randomized 80 VD patients into two groups. The treatment group received *i.v*. administration of gastrodin (400 mg/d), while the control group received i.v. administration of piracetam (800 mg/d). After 4 weeks, the mini mental state examination (MMSE) score was significantly improved in the treatment group (*P* < 0.05), while the control group did not show significant improvement (*P* < 0.05). The result indicated that gastrodin was effective in ameliorating the cognitive impairment in VD patients. He and Wang (2014) investigated the effect of gastrodin combined with donepezil for the treatment of VD. Forty-eight senile VD patients were allocated into two groups in a randomized way. The control group received donepezil (5 mg/d for 12 months) and the treatment group received donepezil plus gastrodin (100 mg/d for 12 months). The MMSE, ability of daily living (ADL) and Hachinski ischemia scales were used for evaluating the memory and cognitive function as well as life quality. In addition, the EEG performance was also evaluated. The results suggested that the two groups both showed progress in EEG and the scores, and the improvement in the gastrodin group was more prominent (*P* < 0.05). This result indicated that gastrodin might be an effective complementary therapy in improving life quality as well as brain function in VD patients.

#### Other cognitive impairment

Learning and memory functions are related to the activities of neurotransmitters including ACh, DA, 5-HT, and others (Jodar and Kaneto, 1995). The cholinergic neuronal system plays an important role in the learning acquisition process, thus scopolamine (SCOP), a muscarinic antagonist that decreases cholinergic activity, could induce impairment of learning acquisition. Hsieh et al. (1997) evaluated the effect of gastrodin on SCOP-induced rat model, and accessed the cognitive function using the one-trial passive avoidance task. They found that gastrodin failed to prolong the shortened step-though latency induced by SCOP in the retention test in rats. However, in the same study, they also discovered that gastrodin was able to prolong the step-through latency induced by cycloheximide (CXM), a protein synthesis inhibitor impairing memory consolidation, or apomorphine (APO), a DA receptor agonist increasing dopaminergic activity and impairing memory retrieval, at the dose of 50 and 5 mg/kg, respectively. The above results suggested that gastrodin could improve amnesia induced by CXM or APO, but not SCOP in rats. It could be conferred that gastrodin could facilitate memory consolidation and retrieval, but not acquisition.

3, 3′-Iminodipropionitrile (IDPN), a nitrile derivative extensively used in manufacturing industry, has neurotoxicity and could induce cognitive impairment in humans and experimental animals. Although the underlying mechanisms have not been well understood, evidence suggests it could lead to alterations in the 5-HT, DA, and ACh neuronal systems and cause damage to hippocampus. Wang X. et al. (2014) established a rat model of cognitive impairment by IDPN exposure, and discovered that gastrodin could significantly mitigate the IDPN-induced cognitive deficits in the Y-maze test, passive avoidance task and Morris water maze (Wang X. et al., 2014; Wang et al., 2015, 2016). In addition, gastrodin could prevent IDPN-induced decrease of DA, 5-HT, and GABA levels and elevation of DA turnover and 5-HT turnover ratios. Furthermore, gastrodin prevented alterations in DA D2 receptor, DA transporter protein, serotonin transporter (SERT), serotonin 1A (5-HT1A) receptor, and a2 GABAA receptor expressions in the hippocampus of the model. These results suggest that gastrodin treatment may have potential therapeutic values for IDPN-induced cognitive impairments, which was mediated by normalizing the DA, 5-TH, and GABAergic systems.

In recent years, the protective effect of gastrodin on cognitive decline after surgery has drawn much attention. In a RCT, Zhang et al. (2011) investigated the effects of gastrodin on cognitive decline after cardiac surgery with cardiopulmonary bypass. Two-hundred patients undergoing mitral valve replacement surgery were randomized to the test group (100 cases), receiving gastrodin (40 mg/kg) after the induction of anesthesia, and control group (100 cases), receiving saline instead. As a result, cognitive decline occurred in 9% of the patients in the test group and 42% in the control group (*P* < 0.01) at discharge. At 3rd month, cognitive decline was detected in 6% in the test group and 31% in the control group (*P* < 0.01). These results indicated that gastrodin is an effective drug for the prevention of neurocognitive decline in patients undergoing mitral valve replacement surgery with CPB. In another clinical trial, Zheng et al. (2016) investigated the effect of gastrodin on cognitive dysfunction after radical operation of cervical carcinoma under epidural anesthesia. 78 cases of cervical cancer were randomly divided into two groups with 39 cases in the control group and 39 cases in the experimental group. The control group was treated with conventional therapy while the experimental group was treated on the basis of the control group with gastrodin (400 mg/d for a week) after the surgery. The cognitive function was accessed at 1st, 3rd, and 7th day after the surgery by using MMSE score. As a result, cognitive dysfunction was observed in both groups after the surgery, and compared with the control group, the MMSE score of the experimental group was higher. Furthermore, the levels of CD4+, CD8+ and CD4+/CD8+ were also significantly higher, and serum S100β protein concentration was lower in the experimental group (*P* < 0. 05). The results showed that gastrodin could improve the cognitive function of patients with cervical carcinoma radical resection under epidural anesthesia as well as reduce serum S100β protein concentration and improve immunity.

## Concluding remarks

As we reviewed above, gastrodin possesses a broad range of biological activities on CNS *in vivo* and *in vitro* and exhibits beneficial effects in a variety of neurological diseases and psychiatric disorders, including epilepsy, AD, PD, depression, anxiety, cerebral I/R, AD, and other cognitive impairment. Its mechanisms of actions include modulating neurotransmitters, anti-oxidation, anti-inflammation, anti-apoptosis, suppressing microglial activation, regulating mitochondrial cascades, up-regulating neurotrophins, etc. (as shown in Table 4). However, in contrast with the large numbers of preclinical experiments, associated clinical trials are relatively scarce, and the methodological qualities of the existing clinical studies were generally low. Thus, more robust clinical trials are required to confirm the claimed therapeutic activities, and further studies are still necessary to elucidate the detailed pharmacokinetics, toxicity, standardization of preparation, and therapeutic doses in human as well. Nevertheless, this compound should be considered for future development as a multi-target agent for therapeutic application of CNS disorders.

## Author contributions

YL, JG, and GS: designed this work of review; MP, HMe, and QZ: performed the literature search of the databases; YL: wrote the manuscript of this paper; PC, HMa, and YX: revised the manuscript; All authors approved the paper for publication.

### Conflict of interest statement

The authors declare that the research was conducted in the absence of any commercial or financial relationships that could be construed as a potential conflict of interest.
